# Supplementary material for: UNNT: A novel Utility for comparing Neural Net and Tree-based models
Source: PLoS Comput Biol. 2024 Apr 29;20(4):e1011504. doi: 10.1371/journal.pcbi.1011504 (PMC11090265; doi:10.1371/journal.pcbi.1011504)
Supplement: S1 Text — (PDF) [file pcbi.1011504.s001.pdf]

Installation + running instructions

1. Download or git clone UNNT repository from GitHub: `https://github.com/vgutta/UNNT.git`
   - *git clone https://github.com/vgutta/UNNT.git*
   - *cd UNNT*
2. Prerequisites
   (a) Anaconda package manager: Installation instructions for your specific system can be found here
3. Install and activate the relevant Anaconda environment
   (a) NVIDIA GPU
      - *conda env create -f gpu_environment.yml -n UNNT_gpu*
      - *conda env activate UNNT_gpu*

   (b) CPU
      - *conda env create -f environment.yml -n UNNT*
      - *conda activate UNNT*
4. Default usage trains CNN and XGBoost models on NCI60 datasets
   (a) *cd UNNT*
   (b) CPU: *python3 unnt.py*
   (c) GPU: *python3 unnt.py --gpu*
5. Data
   (a) All data used to train models with UNNT must be placed in the **data** folder at the root of this repository. The data necessary to run the default settings using NCI60 datasets are also located in the **data** folder. Provide the name of the file for the configuration parameter **data_file**. Name of file must be in quotes and is case-sensitive. The dataset provided is required to be in csv format.
   (b) Provide data that is cleaned and preprocessed into the final format necessary for training. The columns in the dataset must be features. Provide the **target variable** the models train on by setting the **target_variable** configuration parameter in **/UNNT/tree_config.txt**
6. Configuration files
   (a) In addition to configuration on data necessary for the entire software, **/UNNT/tree_config.txt** also contains XGBoost model parameters.
   (b) **/UNNT/cnn_config.txt** contains the configurations specific for CNN model. Many of these are specific to NCI60 dataset used for demo in this software and thus won't affect the models trained using custom data.
