# Supplementary material for: UNNT: A novel Utility for comparing Neural Net and Tree-based models
Source: PLoS Comput Biol. 2024 Apr 29;20(4):e1011504. doi: 10.1371/journal.pcbi.1011504 (PMC11090265; doi:10.1371/journal.pcbi.1011504)
Supplement: S1 FDA — (PDF) [file pcbi.1011504.s002.pdf]

NSC (ID)	Drug Name
740	METHOTREXATE
750	Busulfan
752	6-THIOGUANINE
755	6-MERCAPTOPURINE
757	Colchicine
762	Nitrogen mustard
1390	Allopurinol
2821	Ferulic acid
2833	phlorizin
3053	Actinomycin D
3061	malacid
3069	Chloramphenicol
3088	Chlorambucil
3090	badil
3590	CALCIUM LEUCOVORIN
4291	Masoprocol
4375	Hydroxychloroquine Sulfate
4722	aloin
5364	cinchonidine
5366	Noscapine
5751	salicin
6396	Thiotepa
8096	Beta-Sitosterol
8806	Melphalan
9706	Triethylenemelamine
9896	diandron
10023	Prednisone
10973	Ethinyl estradiol
12198	Dromostanolone Propionate
13875	Altretamine
14229	Acrichine
17474	eskazine
18509	Aminolevulinic acid
19893	Fluorouracil
19987	Methylprednisolone
20256	uridin
23162	Nandrolone phenpropionate
23759	TESTOLACTONE
24559	Mithramycin
25154	Pipobroman
25953	Disulfiram
26271	Cyclophosphamide

26980	Mitomycin
27640	Floxuridine
32065	Hydroxyurea
32982	Curcumin
33299	bena
34462	Uracil mustard
34521	Dexamethasone Decadron
34936	naganol
35347	biosone
35443	Papaverine Hydrochloride
38721	Mitotane
39661	IDOXURIDINE
45388	DACARBAZINE
45923	Methoxsalen
49842	Vinblastine
53477	hctz
56423	Melatonin
57197	Caffeic acid
59272	r-e-s
59687	Acetalax
62791	Ergosterol
63878	Cytarabine
66847	Thalidomide
67574	Vincristine
71047	lactone
71423	Megestrol acetate
75520	tfdu
77213	Procarbazine
77541	Amuno
78206	Cyclobenzaprine Hydrochloride
79037	Lomustine
79269	Vistonuridine
79389	Clofibrate
80998	Cortivazol
81549	Penicillamine
82151	Daunorubicin
82174	Nalidixic Acid
83142	Daunorubicin
85442	Amiodarone hydrochloride
85998	STREPTOZOCIN
88536	Calusterone
89201	Estramustine
90636	Vinblastine

92339	Fluphenazine
92859	Arsenic trioxide
93819	ergenyl
94600	CAMPTOTHECIN
95100	Digoxin
100880	Camptothecin
102816	AZACITIDINE
103805	ancotil
105014	Cladribine
105024	Mithramycin
105823	Acadesine
109229	Asparaginase
109500	Cromolyn sodium
109724	Ifosfamide
109929	Asparaginase
111180	Acetylcysteine
113891	Mesna
113926	rifa
117186	Acetalax
118218	Fludarabine
119875	Cisplatin
122758	Isotretinoin
122819	Teniposide
123127	Doxorubicin
124463	Fludarabine
125066	Bleomycin
125973	Paclitaxel
127716	DECITABINE
129185	melbex
134727	Mitomycin
138783	Bendamustine
141540	Etoposide
141549	M-AMSA
141633	Homoharringtonine
141993	Minocycline Hydrochloride
143020	Mithramycin
145668	Ancitabine hydrochloride
148958	Tegafur
150817	nysert
154948	M-AMSA
156303	M-AMSA
157035	Parthenolide
163039	Ribavirin

163661	3'-Deoxy-2'-thymidinene
169434	florid
169780	Dexrazoxane
170984	Pimozide
177023	Levamisole hydrochloride
180973	Tamoxifen
184849	Mebendazole
208734	aclarubicin
218321	PENTOSTATIN
226080	RAPAMYCIN
227189	ALOIN
241240	Carboplatin
243115	Econazole nitrate
246131	Valrubicin
248616	Spectinomycin Hydrochloride
249992	M-AMSA
256439	Idarubicin
256857	dolgit
256942	Epirubicin
257473	Clotrimazole
265489	Bestatin
266046	Oxaliplatin
279836	MITOXANTRONE
287459	Cytarabine
290193	ciclosporin
292810	Prazosin Hydrochloride
296961	Amifostine
299589	Domperidone
301739	Mitoxantrone
312887	Fludarabine
315851	Bupropion Hydrochloride
317629	Ketoconazole
321521	auranofin
337250	dfmo hcl
352122	TRIMETREXATE GLUCURONATE
362856	Temozolomide
367746	Pentagastrin
369100	Imiquimod
407305	Aloin
409962	Carmustine
526598	Mithramycin
527017	Amphotericin B
602670	timazid

606170	Zalcitabine
606698	Rapamycin
606869	Clofarabine
608000	Tamibarotene
608210	Vinorelbine
609699	Topotecan
612049	Didanosine
613327	Gemcitabine
613680	Toremifene
614826	Bisacodyl, active ingredient of Viraplex
615296	Haloperidol
616348	Irinotecan
620107	PENTAMIDINE ISETHIONATE
628503	Docetaxel
630176	Depsipeptide
632307	Cetuximab
633770	Brivudine
633781	Lovastatin
633782	Simvastatin
639186	Raltitrexed
641530	NEVIRAPINE
648766	Trabectedin
652104	Mifepristone
656576	Midostaurin
661749	Bergenin
664312	Rilmenidine
665970	Artemether
666076	Piroxicam
673596	7-Ethyl-10-hydroxycamptothecin
676879	Phenothiazine, 2-azido-10-[4-(4-methyl-1-piperazinyl)butyl]-, difumarate
677411	leflunomide
681239	Bortezomib
683863	Irofulven
683864	Temsirolimus
685524	CYTISINE
685701	Flurbiprofen
686673	Nelarabine
688097	Trastuzumab
693184	norvir
697855	Nitazoxanide
697979	Denileukin Diftitox Ontak
698037	Pemetrexed
701852	Vorinostat

702294	Estramustine
706363	Arsenic trioxide
707389	Eribulin mesilate
712571	Artesunate
712807	Capecitabine
713563	Exemestane
715055	Gefitinib
718781	Erlotinib
719276	Fulvestrant
719344	Anastrozole
719345	Letrozole
719627	Celecoxib
721075	claritin
721517	Zoledronate
722664	Nelfinavir
724772	Sorafenib
726630	Belinostat
727071	Ofloxacin
727989	Lapatinib
728073	Irinotecan
732517	Dasatinib
733504	Everolimus
737754	Pazopanib
741078	Selumetinib
741485	Finasteride
743414	Imatinib
744009	Sildenafil
745750	Lapatinib
746847	Leuprolide
747167	Nelfinavir
747599	Nilotinib
747856	Olaparib
747971	Sorafenib
747972	Lenalidomide
747973	Ixabepilone
747974	Raloxifene
749226	Abiraterone
749227	Abiraterone
750690	Sunitinib
750691	Afatinib
751548	salinomycin
752782	Pazopanib
753686	Olaparib

754143	Depsipeptide
754230	pralatrexate
754355	Niraparib
754363	Plerixafor
754771	Itraconazole
755384	Pemetrexed
755389	Bosutinib
755400	Masitinib
755401	Golotimod
755404	Flurbiprofen
755605	Enzalutamide
755606	Cediranib
755764	Fluphenazine
755767	Tacrolimus
755768	Fluphenazine
755809	Vismodegib
755841	Actinomycin D
755871	Amphotericin B
755880	Mitomycin
755980	Lenvatinib
755984	Pralatrexate
755985	Nelarabine
755986	Vismodegib
756643	KX-01
756644	Rucaparib
756645	Crizotinib
756647	Quizartinib
756655	Bortezomib
756656	MK-2206
756659	Intedanib
756661	Chlorpromazine
756689	Chlorpromazine
756700	Clotrimazole
756702	COLCHICINE
756714	Cytarabine
756717	DAUNORUBICIN
756738	DIGOXIN
757027	ETHINYL ESTRADIOL
757036	Fluorouracil
757050	badil
757087	Nitrogen mustard
757098	Melphalan
757119	florid

757120	Minocycline Hydrochloride
757306	malacid
757307	Acrichine
757345	Tamoxifen
757348	6-Thioguanine
757363	TYROTHRICIN
757369	eskazine
757384	Vinblastine
757400	pentamidine isethionate
757424	melbex
757425	OUABAIN
757436	Cabozantinib
757437	Salinomycin
757439	Neratinib
757441	Axitinib
757442	Intedanib
757443	Finasteride
757804	Etoposide
757816	Fluphenazine
757838	Mebendazole
757854	Pimozide
757882	Econazole Nitrate
758007	Tivozanib
758183	MONENSIN SODIUM
758186	Azacitidine
758194	ciclosporin
758230	Floxuridine
758244	tepotinib
758246	Trametinib
758247	Palbociclib
758252	Carfilzomib
758253	Homoharringtonine
758254	Ixazomib citrate
758255	Teniposide
758423	M-AMSA
758487	Ponatinib
758612	Bleomycin
758645	Paclitaxel
758662	Lovastatin
758664	Rapamycin
758667	Teniposide
758671	ANCITABINE HYDROCHLORIDE
758682	ARTENIMOL

758706	Simvastatin
758774	Belinostat
758896	Fluvastatin
759155	Doxorubicin
759169	tioconazole
759174	Vincristine
759178	Pipamperone
759195	Epirubicin
759224	Idelalisib
759228	IDEBENONE
759263	Topotecan
759274	ARSENIC TRIOXIDE
759560	AMIODARONE HYDROCHLORIDE
759614	6-Mercaptopurine
759817	artesunate
759850	Docetaxel
759852	Vorinostat
759856	Gefitinib
759857	Clofarabine
759877	Dasatinib
759878	Irinotecan
760057	nitazoxanide
760087	VINORELBINE
760443	Copanlisib
760764	Voreloxin
760766	Vandetanib
761068	Cabozantinib
761190	Panobinostat
761191	brigatinib
761385	Sonidegib
761386	Sonidegib
761388	Plerixafor
761389	Raloxifene
761431	Vemurafenib
761432	Cabazitaxel
761910	Ibrutinib
762523	Elvitegravir
763371	Ruxolitinib
763932	Regorafenib
764040	Alectinib
764042	ARRY-162
764134	Dabrafenib
764487	NVP-BGJ398

764581	ARRY-380
764778	Ivacaftor
764821	Alectinib
765396	BMN-673
765694	Bosutinib
765888	Dacomitinib
765974	BYL-719
766118	Teriflunomide
766270	ABT-199
766907	Ixazomib
767125	BMN-673
767600	Fedratinib
768068	Cobimetinib (isomer 1)
768069	Cobimetinib (isomer 1)
768073	LY-2835219
771649	ARN-509
772469	IPI-145
773149	nitisinone
774769	NMS-E628
775351	Pomalidomide
775772	PF-04449913
776422	LDK-378
777109	E-7438
777193	LDK-378
777878	TABRECTA
778304	Encorafenib
778590	Cobimetinib (isomer 1)
778909	LEE-011
779217	Osimertinib
779405	CO-1686
780108	PF-06463922
780203	selinexor
781556	JNJ-42756493
782351	tofacitinib
785497	INCB-024360
785570	LOXO-101
787457	brigatinib
787846	gilteritinib
788120	Enasidenib
788948	Vistonuridine
789102	ivosidenib
789300	PLX-3397
789925	ABL-001

791164	Acalabrutinib
792848	olmutinib
797937	Sulfatinib
799318	zanubrutinib
800405	umbralisib
801082	BLU-285
809693	Copanlisib
811429	BLU-667
813783	Trabectedin
