# Supplementary figures and images for: UNNT: A novel Utility for comparing Neural Net and Tree-based models
Source: PLoS Comput Biol. 2024 Apr 29;20(4):e1011504. doi: 10.1371/journal.pcbi.1011504 (PMC11090265; doi:10.1371/journal.pcbi.1011504)

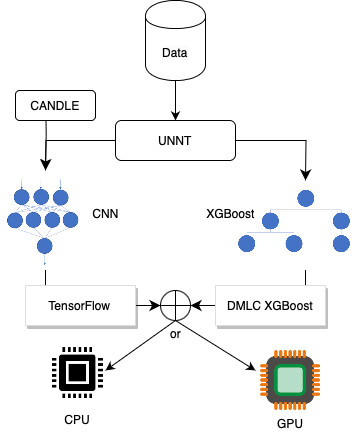

Supplement: S1 Fig — (TIFF) [file pcbi.1011504.s004.tiff]
