# Supplementary material for: UNNT: A novel Utility for comparing Neural Net and Tree-based models
Source: PLoS Comput Biol. 2024 Apr 29;20(4):e1011504. doi: 10.1371/journal.pcbi.1011504 (PMC11090265; doi:10.1371/journal.pcbi.1011504)
Supplement: Attachment — Submitted filename: Review.pdf [file pcbi.1011504.s006.pdf]

Dear PLOS Computational Biology reviewers,

We appreciate your time and effort placed into reviewing our manuscript. Your comments were valuable and lead to the improvements you'll find in the latest version. We considered all your comments and have addressed them as best as we could.

We included reviewers' comments for clarity and our responses to each one can be found immediately following the comment in **bold:**

Reviewer #1: This paper presents a comparison of CNN with XBoost algorithms in the decision of cancer drug responses. It is a nice application and the authors can improve their research as future work. The authors informed the reader that the suggested methods are applicable in the field of chemistry as well. The authors should give information about the research such that they will apply the algorithms for classification or prediction purposes and give necessary references in the suggested algorithms with their applications.

The comment we made regarding the applicability of the methods to chemistry was to indicate that, more than the field of study, the format of the data is what matters for the models. In fact, our datasets also contain chemistry data. The drug descriptor dataset we use for training consists of the chemical features of drugs in the dataset. Each drug is made up of a series of descriptors and they become features in our model. In addition, the gene expression dataset encompasses genomic information regarding each of the cell lines.

When it comes to the application of this method to classification, the nature of the problem, where we predict the AUC value, made us focus on regression. This can easily be converted to a classification problem if the problem allows us to convert the predictor variable to categorical values. In our case, we didn't have that option.

Reviewer #2: Dear Authors,

The manuscript "UNNT: A novel Utility for comparing Neural Net and Tree-based models" presents a comparison between a decision tree-oriented method and deep learning networks in the medical field. The topic is interesting given the profusion of deep learning techniques in scientific works in different areas of application.

Regarding the article, check the punctuation, especially the use of commas. When an acronym is inserted for the first time in the text, its meaning should be presented. Pay attention to long paragraphs as they tend to make reading confusing. I have marked in the

comments of the digital file the passages where the writing should be improved. Please revise and delete repetitive information from the text.

These comments were helpful and the changes we made to address them are in red text. The changes made in response to the comments below are also in red.

I would like your attention to the following recommendations:

1) Lines 59 to 81: cite the bibliographical references that provide support for the information presented.

See references 7 and 8.

2) Line 102: "UNNT splits that data into training, validation, and testing sets" present the percentage for training, validation and testing. Don't forget to detail the method used to draw the samples.

Addressed around line 110.

3) Line 105: "can be set, for both CNN and XGBoost models" . At this point, please note that the configuration of the convolutional network is much more complex than the Random Forest or CARTO methods;

Made a note mentioning that in line 105.

4) Please detail which type of decision tree associated with the XGBoost method was used in the study.

XGBoost used the Gradient Boosted decision tree which is part of a large class of Classification and Regression Tree (CART) method. Gave more details on the difference with the decision tree, especially during splitting, around lines 53-59.

5) Is it possible to run the solution developed in the Cloud? It would also be interesting to point out that a very specific hardware resource was used, which may be beyond the reach of many researchers.

Yes, the instructions provided with the GitHub repository should be sufficient to run UNNT in the cloud as long as Anaconda is installed by the user which is fairly trivial

on most systems. The hardware used in this study can easily be requisitioned on a cloud platform to replicate the same environment as the systems we conducted this study on.

One instance of a unique environment we tested our code on but did not include in the study is a testbed Ookami system at Stony Brook university which contains the ARM-based A64FX processor. It's an accelerator that behaves like the CPU from a software perspective and often requires less code changes than porting to NVIDIA GPUs. This processor is used in the Fugaku system in Japan and was until recently leading the TOP500 list. But we used this system purely for testing and its not a pre

6) Line 135: add as supplementary information at the end of the article.

Thank you, we missed this detail in the submission guidelines. Now in the supplementary materials section.

7) Cite in the text the flowchart of the steps implemented, which is presented at the end of the article.

Mentioned in Design and Implementation section to reference the image in the supplementary materials section.

Note: Upon request from the journal Figure1, referenced in the manuscript, was removed from the supplementary materials document and only uploaded individually.

8) Results: it would be interesting to also present the overall accuracy and precision values. To diversify the presentation of the results, it would be interesting to present graphs, especially the AUC.

We wanted to show accuracy, precision and recall values for the same reasons you specified where these metrics can be graphically represented to readers. However, we didn't see how we could generate those metrics without turning this into a classification model from regression.

9) Still on the results obtained, it would be interesting to compare them with the results obtained by the authors cited in the references.

The CNN model referenced in the study was developed by the authors in the CANDLE study and repositories found in references 1 and 2. The data used in the study was also very similar to one of the original datasets in that study with the changes described in the Data section.

10) Line 196: What is the criterion for defining a sample of 0.5% of the sample set? Please detail what is specified in the FDA approved list.

When we ran grid-search hyperparameter search during training we found 0.9% performed best but 0.5% was also in many of the top results. And many of the top results were extremely close in terms of average accuracy across four test splits. We chose the lower value to prevent overfitting.

mean_fit_tir	std_fit_time	mean_score_	std_score_tir	param_eta	param_max_	param_n_es	param_subsa	params	split0_test_s	split1_test_s	split2_test_s	split3_test_s	split4_test_s	mean_test_s	std_test_sco	rank_test_score
27.368034	0.686501	0.09623184	0.01562998	0.1	6	500	0.9	{'eta': 0.1, 'n	0.85158253	0.85034056	0.85321104	0.85400718	0.85353222	0.85253471	0.00136686	1
46.0722261	0.21317688	0.1039115	0.02420507	0.1	3	2000	0.9	{'eta': 0.1, 'n	0.85150272	0.85035583	0.85319521	0.85387735	0.85333867	0.85245396	0.00131645	2
27.3397424	0.45412173	0.09180837	0.01496663	0.1	6	500	0.7	{'eta': 0.1, 'n	0.85085718	0.84997107	0.8535144	0.85339975	0.85277748	0.85210398	0.00143009	3
25.5185058	0.48514685	0.0891386	0.02830176	0.1	3	1000	0.9	{'eta': 0.1, 'n	0.85089355	0.85000791	0.85238709	0.85303506	0.85279535	0.85182379	0.00117398	4
48.9085422	0.43926963	0.0914125	0.03000385	0.1	6	1000	0.9	{'eta': 0.1, 'n	0.85086827	0.84983369	0.85235036	0.85344368	0.85259265	0.85181773	0.00129351	5
27.161457	0.44841691	0.09108014	0.0205874	0.1	6	500	0.5	{'eta': 0.1, 'n	0.85135592	0.84964445	0.85223235	0.85270859	0.85285068	0.8517584	0.0011791	6
46.4493854	0.30007308	0.07926669	0.02052691	0.1	3	2000	0.7	{'eta': 0.1, 'n	0.8512159	0.84963	0.8520006	0.85267829	0.85317665	0.85174029	0.00124411	7
49.5936742	0.43967168	0.10833135	0.03727071	0.1	6	1000	0.7	{'eta': 0.1, 'n	0.85053302	0.84967968	0.85215917	0.85290745	0.85214993	0.85148585	0.00119042	8
92.4858016	0.88796796	0.13495269	0.00886614	0.1	6	2000	0.9	{'eta': 0.1, 'n	0.85039361	0.8494928	0.85132068	0.8529824	0.85206471	0.85125084	0.00122402	9
25.6194469	0.3401037	0.09067235	0.01848494	0.3	3	1000	0.9	{'eta': 0.3, 'n	0.85066541	0.84905432	0.85168072	0.85265786	0.85204797	0.85122126	0.00126198	10

The FDA approved list is now included in the supplementary materials section and the main reason it was a criteria was because we needed a methodology to reduce the size of the dataset for someone to run this code and the included datasets. FDA approved list is a meaningful method that reduces the size of the data for demonstration purposes.

This concludes our response to the review and feedback we received. Thank you for your time.

Sincerely,
Vineeth Gutta
