# Supplementary material for: UNNT: A novel Utility for comparing Neural Net and Tree-based models
Source: PLoS Comput Biol. 2024 Apr 29;20(4):e1011504. doi: 10.1371/journal.pcbi.1011504 (PMC11090265; doi:10.1371/journal.pcbi.1011504)
Supplement: Attachment — Submitted filename: Second review response.pdf [file pcbi.1011504.s007.pdf]

Dear reviewers,

Thank you for taking the time to give us feedback on our manuscript submission. Please find our responses to your comments below in bold. We included your comments for reference. The new manuscript submission contains a version where changes we made are in red text.

Reviewer #3: This manuscript compares the performance of a convolutional neural network (CNN) and a tree-based method, XGBoost, on a tabular drug response dataset using the software library UNNT developed by the authors.

The limitations of deep learning methods for tabular data and the rigorous comparison of machine learning methods are important topics. The author's work forms a solid foundation for contributing to these areas but would need further development in several areas to make it an improvement beyond existing techniques. I encourage the authors to continue to improve their work in the areas listed below.

Major Issues:

On the machine learning side, the literature review is of limited scope and the references are rather dated. As mentioned above, the difficulties of deep learning on tabular data are an active area of research and the authors need to engage more with recent publications on the subject. Reference [5] in the manuscript is a good starting point, another one could be Grinsztajn, Oyallon, & Varoquaux (NeurIPS 2022).

Thank you for the reference. We read through the paper referenced above and found that their work would nicely complement ours. While they provide references to the datasets and benchmarks, our software, UNNT provides a specific abstraction to test some of the datasets and benchmarks, including the ones from the paper above, on XGBoost and CNN. Their work further reinforces the need for an abstraction like UNNT for researchers to quickly test their own data and see if the conclusions in the studies can be replicated.

The authors frame UNNT as a general tool for the comparison of deep learning models and tree-based models on tabular data but in its current form only two models, CNN and XGBoost, are available. Supporting a greater variety of models, for example deep learning methods specifically designed for tabular data (see e.g. the review of Borisov et al. (IEEE TNNLS 2022)), would make UNNT more

useful for other researchers. The same applies to the availability of evaluation metrics.

Related to the two comments above, I encourage the authors to state more clearly where they see the added value of using UNNT instead of working directly with the machine learning frameworks it is built on. These frameworks already provide many of the features the authors list under Future Directions in their manuscript. It is thus necessary to thoroughly justify why UNNT is needed.

Thank you for this feedback. One of the added values of UNNT is to support domain scientists to test their data against multiple paradigms of ML models and choose the most effectively optimized model that suits their data best. By supporting these domain scientists with our software UNNT, we are facilitating them *(referring to our work in close collaboration with scientists from the National Cancer Institute (NCI))* with easy-to-access software. This approach would allow these users to seamlessly test their datasets with multiple models across frameworks overcoming their barrier to deeply learn about these frameworks. To this end, we believe our work is complementing the NeurIPS 2022 paper. The other goal is to be able to monitor the state-of-the-art in this space to iterate UNNT and improve its capability so domain scientists can continue to advance their science. Without software like UNNT, domain scientists would experience more friction to test their datasets before being able to arrive at some of the conclusions in their paper thus increasing their time-to-solution.

We have updated our manuscript to make the above points clear to the readers. Please find the changes easily in the manuscript with red text to denote changes around lines 33-50 and 108-115.

As much of the model evaluation is based on the R2 score, a more thorough discussion of the applicability of this score to non-linear regression models is required. The limitations of R2 for non-linear models should be discussed openly. This includes the difference in interpretation in the linear and non-linear case. I would recommend not to use “R2 error” but rather “R2 score” or simply “R2”.

This is a valid point and thank you for pointing this out. We fixed the manuscript to refer to it as R2 score and added a disclaimer that the choice of R2 was data dependent for our study. Please find this change in page 6 lines 2016-219.
